# Cytoplasm affects grain weight and filled-grain ratio in *indica *rice

**DOI:** 10.1186/1471-2156-12-53

**Published:** 2011-06-01

**Authors:** Dayun Tao, Peng Xu, Jiawu Zhou, Xianneng Deng, Jing Li, Wei Deng, Jiangyi Yang, Guifeng Yang, Qiong Li, Fengyi Hu

**Affiliations:** 1 Food Crops Research Institute, Yunnan Academy of Agricultural Sciences, Kunming 650205, China

## Abstract

**Background:**

Cytoplasmic effects on agronomic traits -involving cytoplasmic and nuclear genomes of either different species or different cultivars - are well documented in wheat but have seldom been demonstrated in rice (*Oryza sativa *L.). To detect cytoplasmic effects, we introgressed the nuclear genomes of three *indica *cultivars - Guichao 2, Jiangchengkugu, and Dianrui 449 - into the cytoplasms of six *indica *cultivars - Dijiaowujian, Shenglixian, Zhuzhan, Nantehao, Aizizhan, and Peta. These 18 nuclear substitution lines were evaluated during the winter season of 2005 in Sanya, Hainan, China, and during the summer season of 2006 in Kunming, Yunnan, China. The effects of 6 cytoplasm sources, 3 nucleus sources, 2 locations and their interactions were estimated for plant height, panicle length, panicle number per plant, spikelet number per panicle, grain weight, filled-grain ratio, and yield per plot.

**Results:**

For five of the seven traits, analysis of variance showed that there were no significant cytoplasmic effects or interactions involving cytoplasmic effects. The effect of cytoplasm on 1000-grain weight was highly significant. Mean 1000-grain weight over the two locations in four of the six cytoplasms clustered close to the overall mean, whereas plants with Nantehao cytoplasm had a high, and those with Peta cytoplasm a low mean grain weight. There was a highly significant three-way interaction affecting filled-grain ratio. At Sanya, cytoplasms varied in very narrow ranges within nuclear backgrounds. Strong cytoplasmic effects were observed only at Kunming and in only two of the three nuclear backgrounds; in the Jianchenkugu nuclear background, there was no evidence of strong cytoplasmic effects at either location. In the Dianrui 449 and Guichao 2 nuclear background evaluated at Kunming, filled-grain ratios of the six cytoplasms showed striking rank shifts

**Conclusions:**

We detected cytoplasmic variation for two agronomically important traits in *indica *rice. The cytoplasm source had a significant effect on grain weight across the two experimental locations. There was also a significant cytoplasmic effect on filled-grain ratio, but only in two of three nuclear background and at one of the two locations. The results extend our previous findings with *japonica *rice, suggesting that the selection of appropriate cytoplasmic germplasm is broadly important in rice breeding, and that cytoplasmic effects on some traits, such as filled-grain ratio, cannot be generalized; effects should be evaluated in the nuclear backgrounds of interest and at multiple locations.

## Background

Reductions in genetic diversity are of major concern to breeders, geneticists, and the agricultural community in general. In many crops, genetic improvement is usually associated with reduced genetic diversity in the gene pools used to develop the new cultivars, despite the fact that genetic uniformity is believed to increase the potential vulnerability of the crop to biotic and abiotic stresses [1].

The genetic base of cultivars of rice is narrow because of the long history of domestication and genetic improvement. Pedigree analysis indicates that the genetic diversity of *indica *irrigated rice in China depends on a genetic core derived from the varieties Aizizhan, Nantehao, Shenglixian, Peta, and Dijiaowujian [2]. Between three and six sources of genetic material per location have used by breeders of *japonica *rice cultivars in China [2,3], Japan [4], Brazil [1], Senegal [5], and the United States [6], with the cultivars Reimei and Xinan 175 being used as source material at more than one location.

The cytoplasmic genetic base of improved cultivars of rice is also narrow. The WA (wild abortion) cytoplasm makes rice plants male sterile, facilitating the production of cultivars of hybrid rice [7], which explains why 90% of Chinese cultivars of hybrid rice have WA cytoplasm [2]. The situation is similar for cultivars of *indica *rice: most IR varieties--developed by the International Rice Research Institute (IRRI)--trace to the same maternal parent, Cina, implying that components of their cytoplasm are the same [8]. Cina, which has same cytoplasm as the cultivar Peta, is the ultimate maternal parent of 75% of the new (post-IR8) varieties in Sri Lanka, 74% of those in Indonesia, 62% of those in Bangladesh, 60% of those in Korea, 50% of those in the Philippines and 25% of those in Thailand [9]. Eight out of 11 common irrigated varieties in Latin America in the late 1980 s had Cina as a maternal source [10]. Cytoplasms of Aizizhan, Nantehao, Shenglixian, and Cina accounted for 66% of the cytoplasm for 529 *indica *cultivars developed between 1950 and 1984 in southern China [11]. Among 40 diversified cytoplasm resources, cultivar 63-83 was the cytoplasm donor to 60% of upland rice varieties released by the Research Institute for Tropical Agriculture and Food Crops (IRAT) in China [5]. Four of the five main sources of nuclear genetic material in U.S. rice cultivars -- Rexoro, Caloro, Colusa, and Blue Rose -- were also used as core cytoplasm sources in the U.S. [12].

In wheat, another crop with a narrow germplasm base narrowed by centuries of breeding, cytoplasmic effects have been demonstrated using both alloplasmic sources (from related species) [13,14] and euplasmic cytoplasm (from other wheat cultivars) [15-17]. Using reciprocal crossing, maternal effects have been reported for several traits in rice, including low temperature tolerance [18], grain weight [19,20], protein content [21,22], chalkiness [23], milling quality traits [24], panicle number [25], plant regeneration rates [26], hybrid vigor [27,28], and crossability [29].

Research on direct effects of rice cytoplasm has mostly been a quest for cytoplasmic male sterility (CMS) and fertility restoration genes [8]. Compared to the amount of research reported on nuclear effects, the cytoplasmic effect on important agronomic traits in rice has been only sparsely studied. The few reports on maternal effects mentioned above used a reciprocal crossing method. However, this method is not the optimal way to dissect independent cytoplasmic effects because the F_1 _progeny of reciprocal crosses inherit nuclear genetic information from both parents. Therefore, in order to isolate a purely cytoplasmic effect, additional crosses using the same maternal lineage must be made [30]. After 10 backcrosses to the male parent of an F_1 _hybrid, the proportion of progeny that are heterozygous at any individual locus is 1 in 1024. In plants developed this way, 99.9% of the nuclear alleles are expected to be derived from the male parent used in the original cross, while the cytoplamic DNA and approximately 0.1% of the nuclear alleles are derived from the female parent used in the original cross. Using recurrent backcrosses to create nuclear substitution lines, we previously reported a purely cytoplasmic effect on yield, width of flag leaf, and low temperature tolerance in *japonica *rice [31]. To estimate true cytoplasmic effects for *indica *rice, 18 BC_10_F_2:3 _lines were constructed by using 6 core parents of *indica *rice as female parents crossed with 3 recurrent male parents. Herein we report the finding that cytoplasm and its interaction with the nuclear genome and environment can affect grain weight and filled-grain ratio in *indica *rice.

## Results and Discussion

### Cytoplasmic effects

Analysis of data from 18 lines representing all combinations of 6 cytoplasms and 3 nuclei and grown at two locations showed highly significant (*P *≤ 0.001) nuclear effects for all seven traits investigated (analyses of variance not shown). The location effect was also highly significant for panicle number per plant, spikelet number per panicle, filled-grain ratio, panicle length, and plant height, but not for 1,000-grain weight (grain weight) and yield. These results indicate that the nuclear genome was a major factor in expression of important agronomic traits, while most traits were also affected by location.

Among the six core *indica *cytoplasms, there were significant (*P *≤ 0.01) genetic differences in 1,000-grain weight (Table 1). Peta cytoplasm averaged across three nuclear backgrounds was associated with a 1.6 g lower 1,000-grain weight than Nantehao cytoplasm averaged across the same three nuclear backgrounds (Table 2). Cytoplasms of Dijiaowujian, Zhuzhan, Shenglixian, and Aizizhan averaged across these three nuclear backgrounds exhibited 1,000-grain weights that were intermediate between and significantly different from Peta (*P *≤ 0.05) and Nantehao (*P *≤ 0.01) (Table 2).

**Table 1:** Analysis of variance for 1,000-grain weight and filled-grain ratio

Source	Degrees of freedom	1,000-grain weight		Filled-grain ratio	
		
		F value	Pr (> F)	F value	Pr (> F)
Replication	3	4.04	0.0092	2.90	0.038
Cytoplasm (C)	5	3.31	0.0081	0.76	0.58
Nucleus (N)	2	263.9	< 0.0001	22.8	< 0.0001
Environment (E)	1	0.23	0.63	13.9	0.0003
C*N	10	1.27	0.26	0.72	0.71
C*E	5	0.50	0.78	0.68	0.64
N*E	2	14.3	< 0.0001	4.77	0.01
C*N*E	10	1.9	0.052	4.03	0.0001
Error (mean square)	105	(2.05)		(120.4)	

**Table 2:** Mean 1,000-grain weights for six cytoplasms averaged across three nuclear backgrounds of *indica *rice

Cytoplasm	1,000-grain weight (g)	Significance tests^a^	
		
		P = 0.05	P = 0.01
Nantehao	25.6	a	A
Dijiawujian	24.9	ab	AB
Zhuzhan	24.8	abc	AB
Shenglixian	24.7	abc	AB
Aizizhan	24.5	bc	AB
Peta	24.0	c	B

^a ^Values followed by the same letter are not significantly different. Lower-case letters indicate significant differences at P = 0.05, upper-case at 0.01

The highly significant effect of cytoplasm on grain weight was consistent with the results of Chandraratna in 1960 [14] and Somrith in 1979 [15], who used the reciprocal crossing method to show that the cytoplasm affects grain weight. Both the nuclear substitution method (ours) and the reciprocal crossing method (Chandraratna's and Somrith's) showed that cytoplasm or cytoplasm-nucleus interactions affected grain weight, which is a major component of yield and grain quality in rice production.

In our experiment, the location effect alone was non-significant for 1,000-grain weight, but there was a highly significant (*P *≤ 0.001) effect due to interaction between location and nuclear effects (Table 1). These results--showing that the change of grain weight in rice was mainly dependent on the effect of both cytoplasm and nucleus, while the location exerted an effect via interaction with the nucleus--were consistent with the conclusions of Pham [32]. This strongly suggests that in future rice breeding, genetic studies, and germplasm resources research, the role of cytoplasm should receive more attention.

There was a highly significant three-way interaction affecting filled-grain ratio (Table 1). Cytoplasm-nucleus interactions were significant at both locations, but the main effect of cytoplasm was significant only at Kunming (Table 3). That led us to dissect further the effect of cytoplasm and its interaction with the nucleus and location.

**Table 3:** Analyses of variance for filled-grain ratio at two locations

Source	Degrees of freedom	Kunming		Sanya	
			
		F value	Pr (> F)	F value	Pr (> F)
Replication	3	1.25	0.30	3.45	0.23
Cytoplasm (C)	5	3.00	0.02	0.25	0.93
Nucleus (N)	2	51.38	< 0.0001	7.18	0.0018
C*N	10	2.77	0.0082	2.59	0.0128
Error (mean square)	105	(21.9)		(80.2)	

### Effects of cytoplasm and interaction with nucleus and location

**Figure 1:**
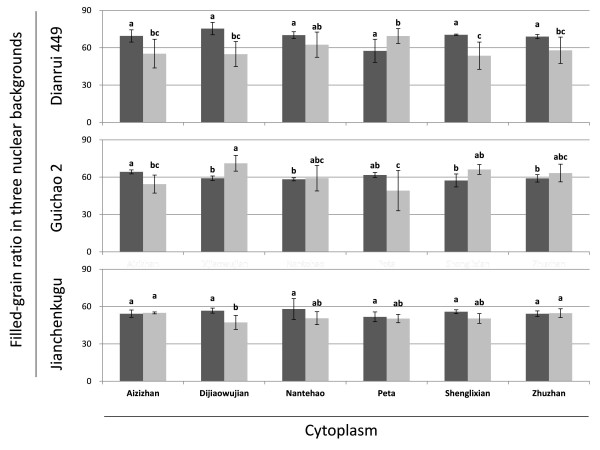
**Mean filled-grain ratios for six cytoplasms in three nuclear backgrounds at two locations**. The left, grey bar in each pair represents the mean in Sanya: the right, black bar represents Kunming. Within each location-nuclear background combination, mean bars labeled with different letters are significantly different at the P = 0.05 level.

Despite the significance of cytoplasmic-nuclear interaction for filled-grain ratio at Sanya, cytoplasms varied in very narrow ranges in each of the three nuclear backgrounds (in Figure 1, the grey, left-hand bar in each pair). Strong cytoplasmic effects were observed only at Kunming, but in only two of the three nuclear backgrounds: Dianrui 449 and Guichao 2 (in Figure 1, the black, right-hand bar in each pair); in the Jianchenkugu nuclear background, there was no evidence of strong cytoplasmic effects at either location.

At Kunming, filled-grain ratios associated with the six cytoplasms showed striking rank shifts in Dianrui 449 and Guichao 2 nuclear backgrounds (Figure 1, top and bottom). The mean for lines with Peta cytoplasm, for example, was in the lowest group of lines with the Guichao 2 nuclear genome, but Peta cytoplasm exhibited the highest filled-grain ratio. Lines with Shenglixian cytoplasm showed the opposite pattern to that of Peta, being in the top group in the Guichao 2 nuclear background while ranking lowest in the Dianrui 449 background.

The cytoplasmic-nuclear interaction based both locations for the rest traits, which were plant height (cm), panicle length(cm), panicle number per plant, spikelet number per panicle, 1,000-grain weight (g) and yield per plot (kg), shown in Additional Files 1, 2, 3, 4, 5, 6 and 7.

### Implications for rice breeding

Cytoplasmic variation represents a largely unexplored genetic resource for *indica *rice breeding programs, and exploitation of all available genetic resources is seen as necessary for avoiding a future cereal "yield plateau" [33]. Increased grain weight is one way to increase sink strength, which itself is a means of increasing cereal yield potential [34]. Although the nuclear effect for grain weight was larger than the cytoplasmic effect in this study (Table 1), nuclear genetic variation for grain weight is small compared with that for other agronomic traits [35]. The six core cytoplasms of *indica *rice were evaluated in this study. Evaluation of germplasm collections may reveal that other cytoplasmic sources not studied herein are superior for increasing *indica *rice grain weight.

Rice breeders are already interested in diversifying the cytoplasmic base of cultivar development programs to reduce the chance of disease epidemics. The vulnerability of cytoplasmic uniformity was illustrated in the classic case of Southern corn leaf blight caused by *Helminthosporium maydis*. An epidemic of the blight in 1970 devastated the U.S. maize crop because a disease-susceptible cytoplasm had been widely used in the development of male-sterile. Rice is similarly vulnerable, as demonstrated by the fact that WA cytoplasm affects the expression of bacterial blight reaction significantly [36] and is more seriously infected by blast isolate 90-2 than are its maintainers [37]. The discovery that cytoplasmic diversity affects other agronomic traits (both positively and negatively) makes it essential that cytoplasms being evaluated for disease resistance and CMS also be carefully evaluated for effects on other traits such as grain weight and filled-grain ratio.

The significant interaction effects we found between cytoplasm and nucleus and/or location have important implications for breeding programs. If elite nuclei are substituted into different cytoplasms to utilize cytoplasmic genes for CMS, disease resistance or agronomic traits, the resulting alloplasmic lines must be thoroughly tested in all nuclear-cytoplasmic combinations at all target locations even if the original elite lines were locally adapted. On the other hand, perhaps cytoplasmic substitution could be a useful method for generating location-specific cultivars from elite lines without disrupting favorable combinations of nuclear genes, including transgenes.

## Conclusions

Compared with nuclear genes, cytoplasmic genes played a modest but statistically significant role in determining grain weight and filled-grain ratio of *indica *rice lines. The cytoplasmic effect for grain weight was significant across locations but the effect on filled-grain ratio was significant only as an interaction with location or as an interaction with nuclei at one location. These results suggest that useful genetic variation for agronomic traits may be present in the *indica *cytoplasmic gene pool. The unpredictable--but not surprising--interaction of cytoplasm source with nucleus source and location for one trait in this study suggests that breeders should beware of assuming that a particular cytoplasm will be equally adaptive in all locations or that nuclei and cytoplasms can be substituted freely without extensive testing in each location. On the other hand, nuclei with elite combinations of genes for yield, grain quality or other traits could be substituted into a number of distinct cytoplasms in the hopes of finding combinations better adapted for particular locations than the original nucleus donor cultivar.

## Methods

### Materials

Six major cultivars of *indica *rice -- Dijiaowujian, Shenglixian, Zhuzhan, Nantehao, Aizizhan, and Peta -- were used as female parents in crosses with 3 other *indica *rice cultivars: Guichao 2, Jiangchengkugu, and Dianrui 449. The latter 3 cultivars were used as recurrent parents in 10 backcrosses to form BC_10_F_1 _nuclear substitution lines, which were then selfed to produce the BC_10_F_2 _generation. Plants from this generation were selfed and the seeds were bulked for planting four replicates at two locations.

### Experiment

Eighteen nuclear substitution lines of BC_10_F_2:3 _and their parents were sown on 5 November, and transplanted on 26 November, 2005, in Sanya, Hainan (November-April) for agronomic evaluation in the winter cropping season at a tropical location. Parental cytoplasm-nucleus combinations were excluded, in order to maintain a factorial design of six cytoplasmic genomes by three nuclear genomes. The traits measured were yield per plot (kg), 1,000-grain weight (g), panicle number per plant, spikelet number per panicle, filled-grain ratio (%), panicle length (cm), and plant height (cm). Plot size was 1.5 m × 2.5 m. Plant spacing was 15 cm between plants within a row and 25 cm between rows. The design was a randomized complete block with four replicates. The experiment was repeated at the Yunnan Academy of Agricultural Sciences, Kunming, from March to September 2006 for agronomic evaludation at a subtropical location. Cultural and management practices, including fertilizer applied (15 kg ha^-1 ^of N, P, and K), were similar for both experiments at Sanya and Kunming.

### Statistical analysis

Analysis of variance of the 6 × 3 factorial genetic design was used in order to determine in a single comprehensive analysis which among the seven traits were influenced by differences in the cytoplasmic genome and whether those effects depended on the nuclear background, the environment, or both. That preliminary information allowed us then to examine in more detail the differences and rankings of individual cytoplasms where significant differences occurred. Except for yield per plot, all data were from observations on 10 individuals selected randomly from each plot, and the plot mean was used in the analysis of variance. Fixed model analyses using type III sums of squares in SAS PROC GLM [38] were used to detect effects of nucleus, cytoplasm, and interaction between cytoplasm, nucleus, and location. For filled-grain ratio, arscin transformation of the ratio was performed before the analysis. If the three-way interactional effect among cytoplasm, nucleus, and location was significant for traits investigated, the least-squares means (LS-means) of fixed effects was employed to analyze the cytoplasmic effects within each location and nucleus. Duncan's multiple range test was used for multiple comparisons of the effect of cytoplasm and cytoplasm interaction with nucleus and location.

## Authors' contributions

DT participated in designing the experiment and in drafting the manuscript. PX made some of the nuclear substitution lines and carried out the experiment in Sanya, Hainan. JZ made some of the nuclear substitution lines and carried out the experiment in Kunming, Yunnan. XD carried out the experiment in Sanya, Hainan. JL, assistant to JZ, carried out the experiment in Kunming, Yunnan. WD, assistant to PX and XD, carried out the experiment in Sanya, Hainan. GY made some of the nuclear substitution lines. JY made some of the nuclear substitution lines. QL, assistant to JW, carried out the experiment in Kunming, Yunnan. FH made some of the nuclear substitution lines, participated in designing the experiment, analyzed the data, and participated in drafting the manuscript. All authors have read and approved the final manuscript.

## Supplementary Material

Additional file 1 **Agronomic-trait data on which Figure 1 and Additional Figures 1 through 6 are based**. Means for seven traits expressed by backcross lines representing all combinations of three nuclear and six cytoplasmic genomes at two locations with four replications at each location. Click here for file

Additional file 2 **Mean plant height of 18 nuclear-cytoplasm combinations in *indica *rice**. Means and standard errors for plant height recorded at two locations, Sanya and Kunming, China, from 18 backcross lines of *indica *rice representing all combinations of three nuclear and six cytoplasmic genomes. Click here for file

Additional file 3 **Mean panicle length of 18 nuclear-cytoplasm combinations in *indica *rice**. Means and standard errors for panicle length recorded at two locations, Sanya and Kunming, China, from 18 backcross lines of *indica *rice representing all combinations of three nuclear and six cytoplasmic genomes. Click here for file

Additional file 4 **Mean number of panicles per plant of 18 nuclear-cytoplasm combinations in *indica *rice**. Means and standard errors for number of panicles per plant recorded at two locations, Sanya and Kunming, China, from 18 backcross lines of *indica *rice representing all combinations of three nuclear and six cytoplasmic genomes. Click here for file

Additional file 5 **Mean number of spikelets per panicle of 18 nuclear-cytoplasm combinations in *indica *rice**. Means and standard errors for number of spikelets per panicle recorded at two locations, Sanya and Kunming, China, from 18 backcross lines of *indica *rice representing all combinations of three nuclear and six cytoplasmic genomes. Click here for file

Additional file 6 **Mean 1000-grain weight of 18 nuclear-cytoplasm combinations in *indica *rice**. Means and standard errors for 1000-grain weight recorded at two locations, Sanya and Kunming, China, from 18 backcross lines of *indica *rice representing all combinations of three nuclear and six cytoplasmic genomes. Click here for file

Additional file 7 **Mean yield per plot of 18 nuclear-cytoplasm combinations in *indica *rice**. Means and standard errors for yield per plot recorded at two locations, Sanya and Kunming, China, from 18 backcross lines of *indica *rice representing all combinations of three nuclear and six cytoplasmic genomes. Click here for file

## References

[B1] Chatel M Guimaraes E Ospina Y Borrero J Piggin C, Courtois B, Schmi V Improvement of upland rice using gene pools and populations with recessive male-sterility gene Upland rice research in partnership 1996 International Rice Research Institute, P.O. Box 933, Manila, Philippines 284 298

[B2] Lin SC Min SK Rice varieties and their genealogy in China 1991 Shanghai Science and Technology Press, Shanghai, China

[B3] Yang SX Xiong ZM, Cai HF, Min SK, Li BC Rice in Yunnan Rice in China 1992 China Agricultural Science and Technology Press, Beijing 421 436

[B4] Kikuchi F Semidwarfing genes of high yielding rice varieties in Japan Rice Genetics 1986 International Rice Research Institute, P. O. Box 933, Manila, Philippines 85 295

[B5] Hu FY Tao DY Yang GF Yang JY Poisson C, Rakotoarisoa J Genealogical analysis of IRAT upland rice varieties Rice cultivation in highland areas, Proceedings of the CIRAD conference held at Antananarivo, Madagascar 1997 CIRAD, Montpellier, France 181 184

[B6] Dilday RH Contributions of ancestral lines in the development of new cultivars of rice Crop Science 1990 30 905 911 10.2135/cropsci1990.0011183X003000040030x

[B7] Yuan LP The concept of the strategy of hybrid rice breeding Hybrid Rice 1988 2 1 3

[B8] Khush GS Virk PS IR Varieties and Their Impact 2005 Los Banos (Philippines): International Rice Research Institute 163

[B9] Hargrove TR Coffman WR Cabanilla VL Genetic interrelationships of improved rice varieties in Asia IRRI Research Paper Series 23 1979 International Rice Research Institute. P.O. Box 933, Manila, Philippines 34

[B10] CIAT (Centro Internacional de Agricultura Tropical) Rice program 1986-1989 Report, Working document No. 92 1991 CIAT, Cali, Colombia 109 113

[B11] Gu MH Pan XB Li X Genetic analysis of the pedigrees of the improved cultivars in *Oryza sativa *L*Subsp. Hsien in South China* Scientia Agricultura Sinica 1986 1 41 48

[B12] Mackill D McKenzie Smith CW, Dilday RH Origin and characteristics of U.S. rice cultivars Rice Origin, History, Technology, and Production 2003 Hoboken, John Wiley & Sons 87 100

[B13] Ekiz H Konsak CF Nuclear and cytoplasmic control of anther culture response in wheat. I. Analyses of alloplasmic lines Crop Science 1991 31 1421 1427 10.2135/cropsci1991.0011183X003100060005x

[B14] Ekiz H Safi Kiral A Akcin A Simsek L Cytoplasmic effects on quality traits of bread wheat (Triticum aestivum L.) Euphytica 1998 100 189 196 10.1023/A:1018382106978

[B15] Ekiz H Konsak CF Nuclear and cytoplasmic control of anther culture response in wheat. III. Common wheat crosses Crop Science 1991 31 1432 1436 10.2135/cropsci1991.0011183X003100060007x

[B16] Voluevich EA Palilova AN Effect of maternal cytoplasm on resistance to brown rust in bread wheat Tsitologiya I Genetika 1988 22 34 37

[B17] Ozgen M Turet M Avci M Cytoplasmic effects on the tissue culture response of callus from winter wheat mature embryos Plant Cell Tissue and Organ Culture 2001 64 81 84 10.1023/A:1010609603915

[B18] Ratho SN Pradhan SB Cytoplasmically controlled cold tolerance in a cytoplasmic-genetic male sterile line of rice Euphytica 1992 58 241 244

[B19] Chandraratna MF Sakai KI A biometrical analysis of matroclinous inheritance of grain weight in rice Heredity 1960 14 365 373 10.1038/hdy.1960.35

[B20] Somrith B Chang TT Jackson BR International Rice Research Institute, Los Banos, Philippines Genetic analysis of traits related to grain characteristics and quality in two crosses of rice IRRI Research Paper Series 35 1979

[B21] Chang TT Lin FH Diallel analysis of protein content in rice Agronomy Abstracts 1974 65

[B22] Shi CH Xue JM Yu YG Yang XE Zhu J Analysis of genetic effects on nutrient quality traits in *indica *rice Theor Appl Genet 1996 92 1099 1102 10.1007/BF00224055 24166642

[B23] Xu YB Shen ZT Maternal effect on chalkiness in rice kernels RGN 1988 5 111 113

[B24] Shi CH J Zhu J Analysis of seed, cytoplasmic, and maternal genetic effects on rice quality traits IRRN 1995 20 2 4

[B25] Wu HP Studies on the quantitative inheritance of *Oryza sativa *L. I. Diallel analysis of heading time and plant height in F1 progeny Bot Bull Acad Sin 1968 9 2 1 9

[B26] Chu QR Croughan TP Mujeeb-kazi A, Sitch LA, CIMMYT and IRRI, Mexico, D. F Genetics of plant regeneration in anther culture (AC) of rice (*Oryza sativa *L.) Review of advances in plant biotechnology, 1985-88: 2nd International Symposium on Genetic manipulation in Crops 1989 Mexico, and Manila, Philippines 217 227

[B27] Young JB Virmani SS Effects of cytoplasm on heterosis and combining ability for agronomic traits in rice (*Oryza sativa *L.) Euphytica 1990 48 177 188 10.1007/BF00037198

[B28] Virmani SS Heterosis and hybrid rice breeding Monographs on Theoretical and Applied Genetics 1994 International Rice Research Institute, Springer-Verlag 26 30

[B29] IRRI (International Rice Research Institute) Program report for 1993 1994 International Rice Research Institute, P.O. Box 933, Manila, Philippines 131 156

[B30] Roach DA Wulff RD Maternal Inheritance in Plants Annual Review of Ecology and Systematics 1987 18 209 235 10.1146/annurev.es.18.110187.001233

[B31] Tao D Hu F Yang J Yang G Yang Y Xu P Li J Ye C Dai L Cytoplasm and cytoplasm-nucleus interactions affect agronomic traits in japonica rice Euphytica 2004 135 1 129 134

[B32] Pham JL Genetic diversity and intervarietal relationships in rice (*Oryza sativa *L.) in Africa Rice genetics II 1991 International Rice Research Institute, Manila 1099, Philippines 55 65

[B33] Hoisington D Khairallah M Reeves T Ribaut JM Skovmand B Taba S Warburton M Plant genetic resources: What can they contribute toward increased crop productivity? Proc Natl Acad Sci USA 1999 96 5937 5943 10.1073/pnas.96.11.5937 10339521 PMC34209

[B34] Richards RA Selectable traits to increase crop photosynthesis and yield of grain crops Journal of Experimental Botany 2000 51 447 458 10.1093/jexbot/51.suppl_1.447 10938853

[B35] Luo LJ Li ZK Mei HW Shu QY Tabien R Zhong DB Ying CS Stansel JW Khush GS Paterson AH Overdominant Epistatic Loci Are the Primary Genetic Basis of Inbreeding Depression and Heterosis in Rice. II. Grain Yield Components Genetics 2001 158 1755 1771 11514460 10.1093/genetics/158.4.1755 PMC1461757

[B36] Yang RC Susceptibility of A lines and B lines to bacterial blight (BB) IRRN 1987 12 6 7

[B37] Liu KM Wang LS Wei LK Zhu XY Wu QA Reaction of rice male sterile cytoplasm of Wild Abortion type to the infection of *Pyricularia oryzae* Scientia Agricultura Sinica 1992 25 2 92

[B38] SAS Institute SAS/STAT 1996 SAS Institute, Cary, NY

