# Supplementary figures and images for: Cytoplasm affects grain weight and filled-grain ratio in indica rice
Source: BMC Genet. 2011 Jun 1;12:53. doi: 10.1186/1471-2156-12-53 (PMC3118132; doi:10.1186/1471-2156-12-53)

## Slide 1
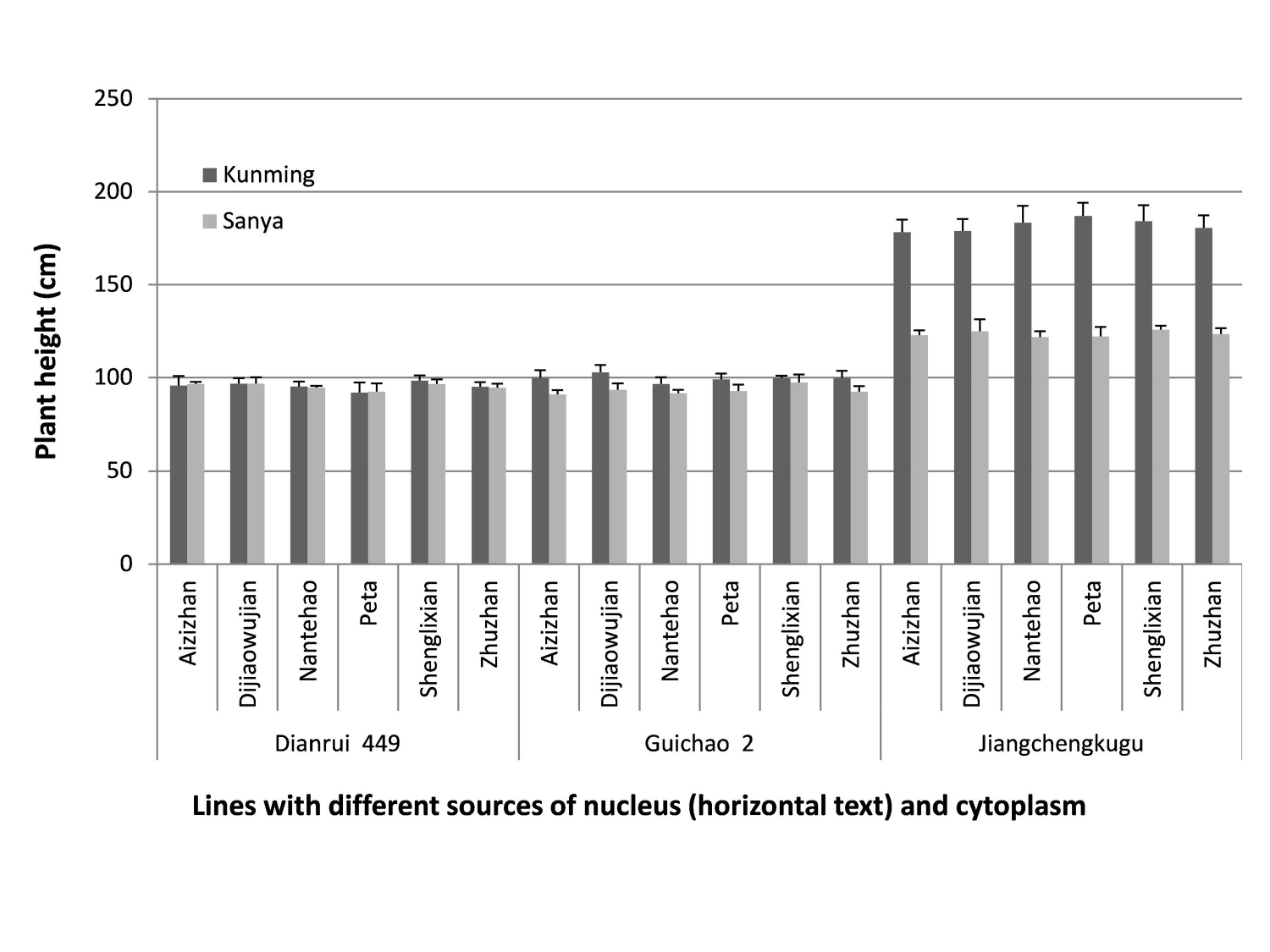

Supplement: Additional file 2 — Mean plant height of 18 nuclear-cytoplasm combinations in indica rice. Means and standard errors for plant height recorded at two locations, Sanya and Kunming, China, from 18 backcross lines of indica rice representing all combinations of three nuclear and six cytoplasmic genomes. [file 1471-2156-12-53-S2.PPT]

## Slide 1
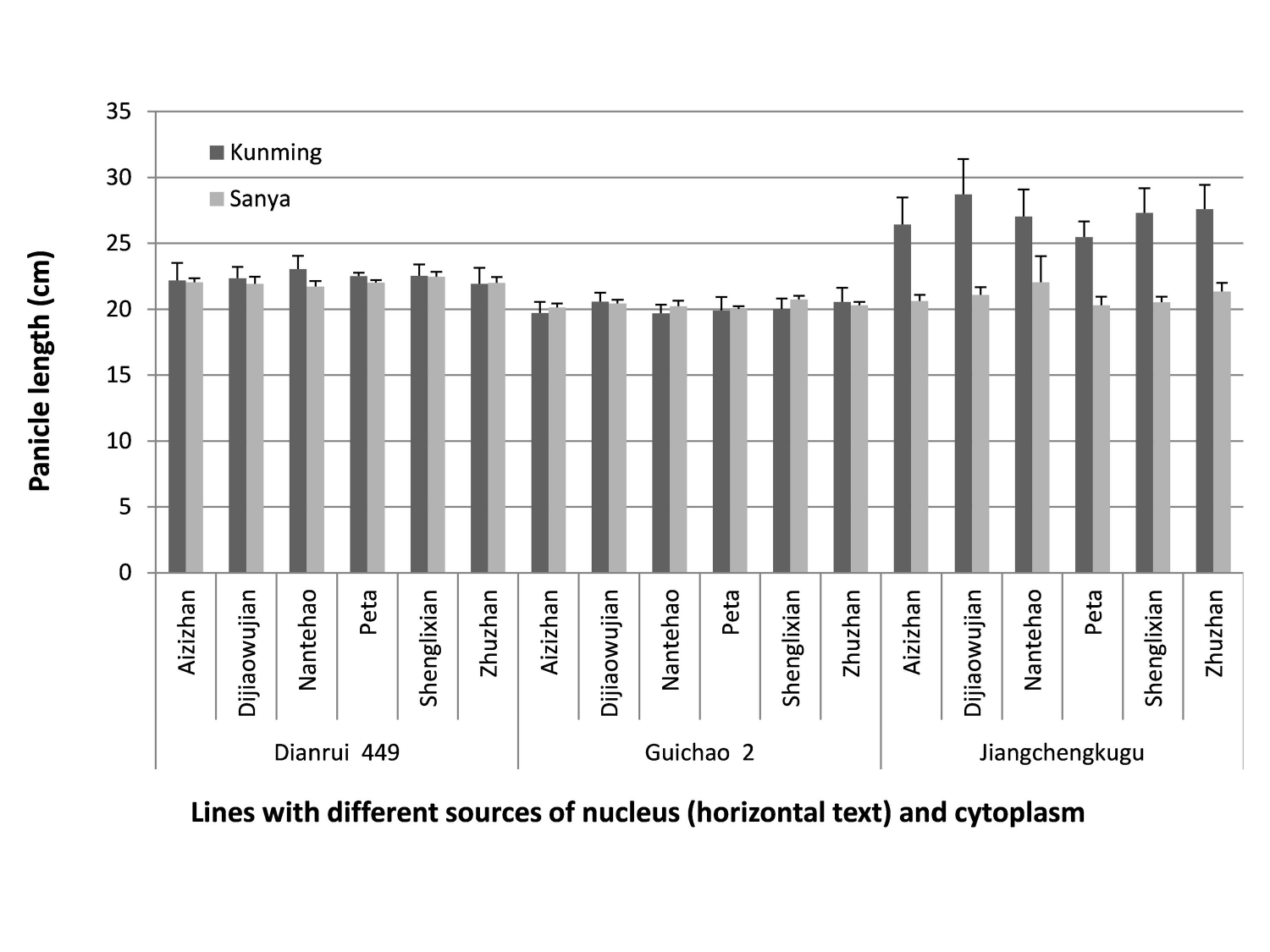

Supplement: Additional file 3 — Mean panicle length of 18 nuclear-cytoplasm combinations in indica rice. Means and standard errors for panicle length recorded at two locations, Sanya and Kunming, China, from 18 backcross lines of indica rice representing all combinations of three nuclear and six cytoplasmic genomes. [file 1471-2156-12-53-S3.PPT]

## Slide 1
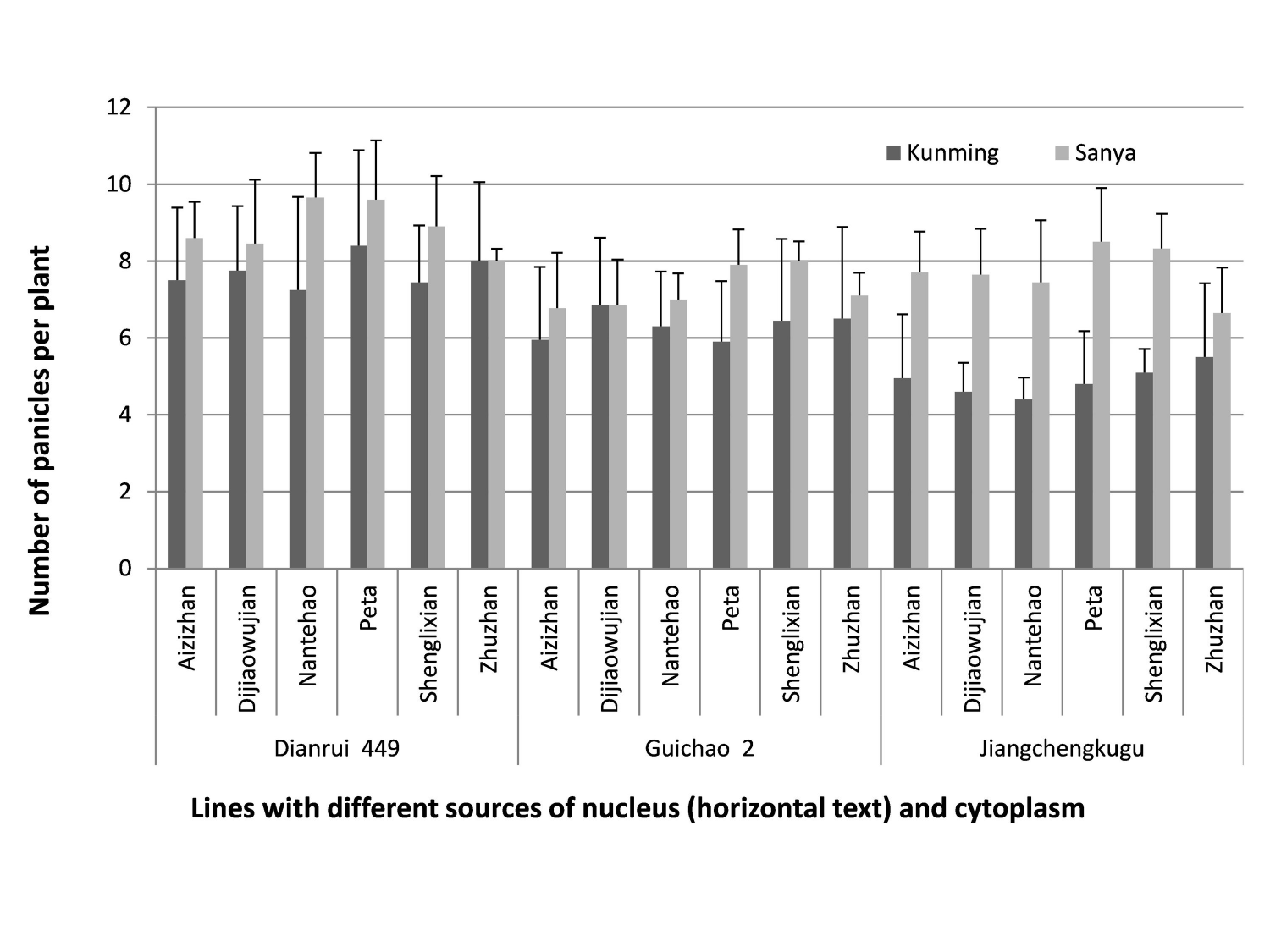

Supplement: Additional file 4 — Mean number of panicles per plant of 18 nuclear-cytoplasm combinations in indica rice. Means and standard errors for number of panicles per plant recorded at two locations, Sanya and Kunming, China, from 18 backcross lines of indica rice representing all combinations of three nuclear and six cytoplasmic genomes. [file 1471-2156-12-53-S4.PPT]

## Slide 1
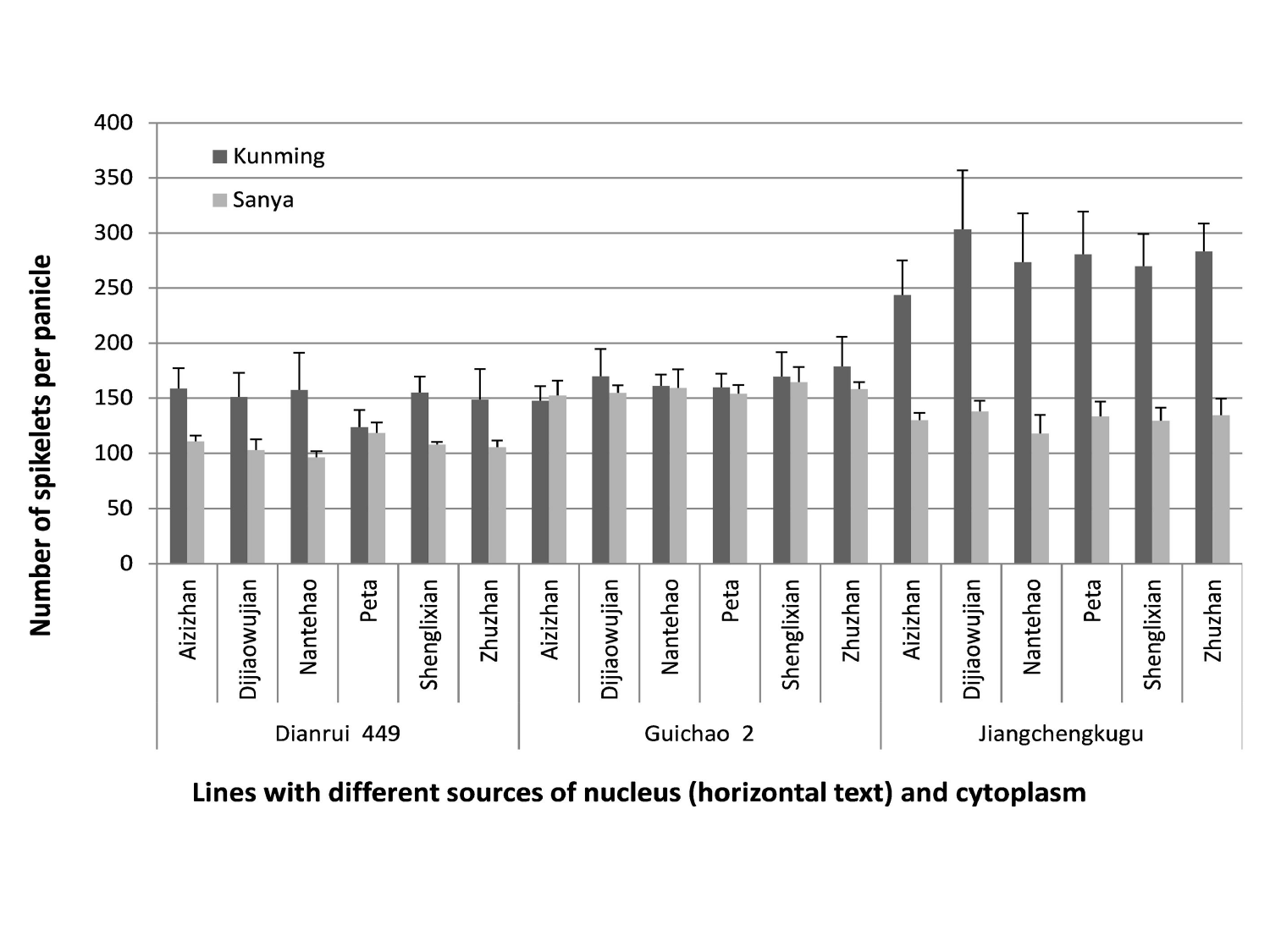

Supplement: Additional file 5 — Mean number of spikelets per panicle of 18 nuclear-cytoplasm combinations in indica rice. Means and standard errors for number of spikelets per panicle recorded at two locations, Sanya and Kunming, China, from 18 backcross lines of indica rice representing all combinations of three nuclear and six cytoplasmic genomes. [file 1471-2156-12-53-S5.PPT]

## Slide 1
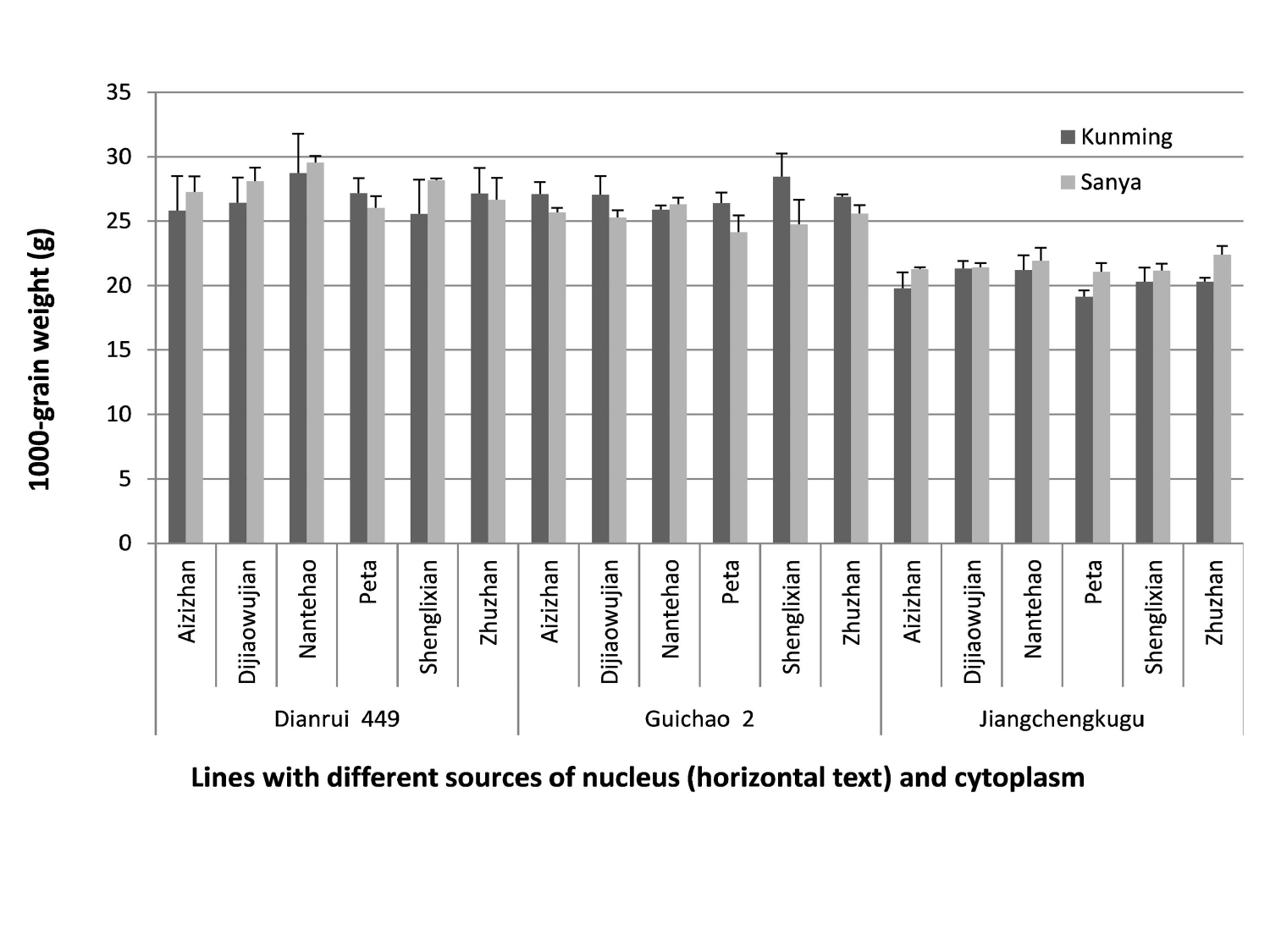

Supplement: Additional file 6 — Mean 1000-grain weight of 18 nuclear-cytoplasm combinations in indica rice. Means and standard errors for 1000-grain weight recorded at two locations, Sanya and Kunming, China, from 18 backcross lines of indica rice representing all combinations of three nuclear and six cytoplasmic genomes. [file 1471-2156-12-53-S6.PPT]

## Slide 1
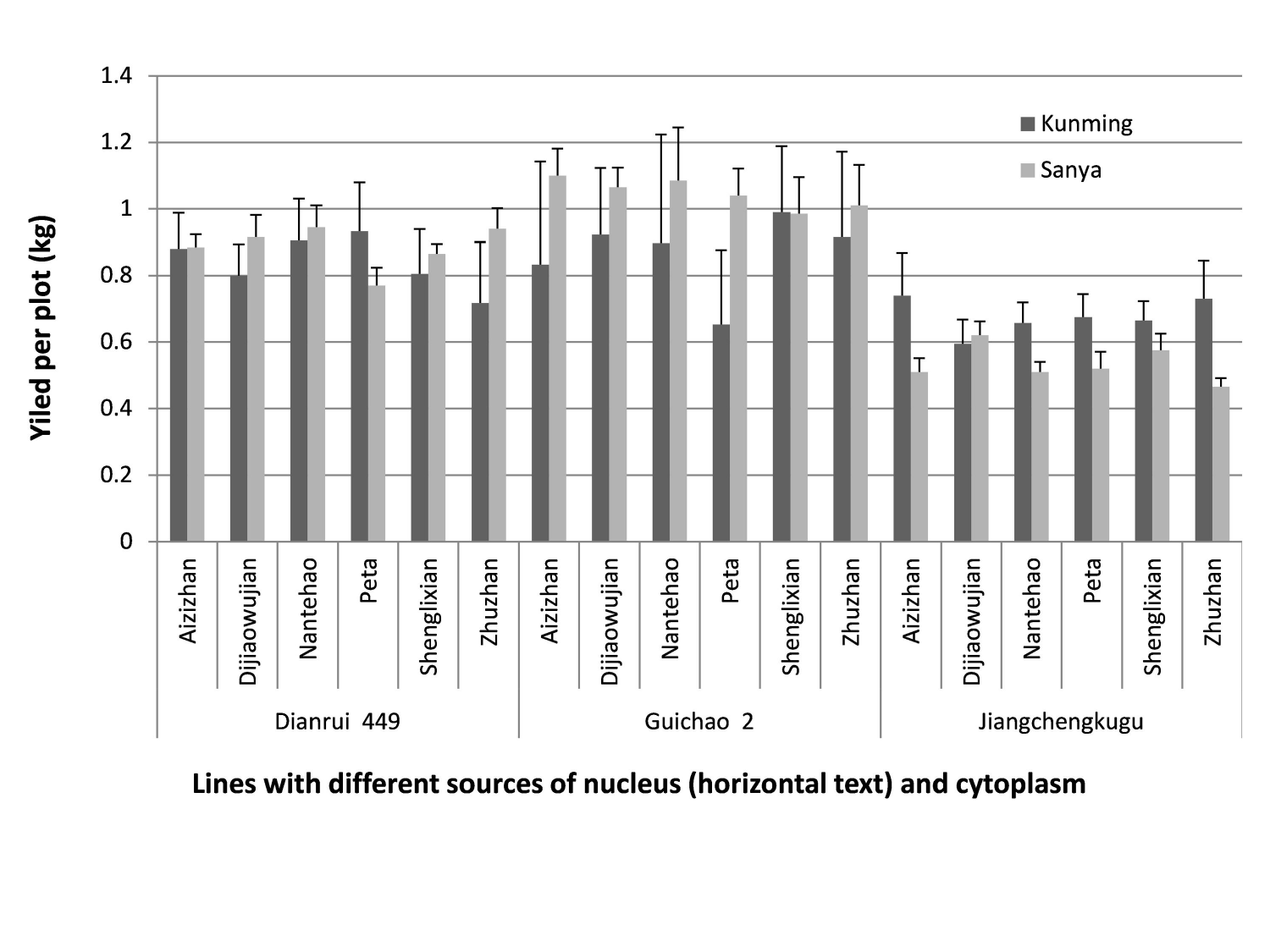

Supplement: Additional file 7 — Mean yield per plot of 18 nuclear-cytoplasm combinations in indica rice. Means and standard errors for yield per plot recorded at two locations, Sanya and Kunming, China, from 18 backcross lines of indica rice representing all combinations of three nuclear and six cytoplasmic genomes. [file 1471-2156-12-53-S7.PPT]
